# Study on characteristic of epileptic multi-electroencephalograph base on Hilbert-Huang transform and brain network dynamics

**DOI:** 10.3389/fnins.2023.1117340

**Published:** 2023-05-03

**Authors:** Xiaojie Lu, Tingting Wang, Mingquan Ye, Shoufang Huang, Maosheng Wang, Jiqian Zhang

**Affiliations:** ^1^School of Physics and Electronic Information, Anhui Normal University, Wuhu, China; ^2^Research Center of Health Big Data Mining and Applications, School of Medicine Information, Wan Nan Medical College, Wuhu, China

**Keywords:** Hilbert-Huang transform, CNN, symbolic transfer entropy, brain network, Kuramoto model

## Abstract

Lots of studies have been carried out on characteristic of epileptic Electroencephalograph (EEG). However, traditional EEG characteristic research methods lack exploration of spatial information. To study the characteristics of epileptic EEG signals from the perspective of the whole brain，this paper proposed combination methods of multi-channel characteristics from time-frequency and spatial domains. This paper was from two aspects: Firstly, signals were converted into 2D Hilbert Spectrum (HS) images which reflected the time-frequency characteristics by Hilbert-Huang Transform (HHT). These images were identified by Convolutional Neural Network (CNN) model whose sensitivity was 99.8%, accuracy was 98.7%, specificity was 97.4%, F1-score was 98.7%, and AUC-ROC was 99.9%. Secondly, the multi-channel signals were converted into brain networks which reflected the spatial characteristics by Symbolic Transfer Entropy (STE) among different channels EEG. And the results show that there are different network properties between ictal and interictal phase and the signals during the ictal enter the synchronization state more quickly, which was verified by Kuramoto model. To summarize, our results show that there was different characteristics among channels for the ictal and interictal phase, which can provide effective physical non-invasive indicators for the identification and prediction of epileptic seizures.

## 1. Introduction

Epilepsy is a neurological disease caused by sudden abnormal hyper-synchronization discharge behavior of neurons in the brain, causing involuntary behavior and seizures. Electroencephalogram (EEG) signals could be used to monitor the electrical activity in the brain. They record the electrical wave changes during brain activity and are the overall reflection of the electrophysiological activities of brain nerve cells on the scalp surface. EEG contains abundant brain information and is one of the means of clinical diagnosis of brain diseases (Proix et al., 2018).

Diagnosis of epilepsy by EEG requires a well-trained clinician or neurophysiologist, however, detecting through artificial intelligence has the potential to improve the quality of medical care by shortening diagnosis time, reducing manual errors, and relieving physician fatigue. Many analyzing and processing techniques of signals have been proposed for studying EEG signals (Shoeibi et al., 2021). The time-frequency analysis methods have attracted the attention of many scholars. Hilbert Huang Transform (HHT) are commonly used to process non-stationary signals (Wu and Huang, 2009; Supriya et al., 2020). Empirical Mode Decomposition (EMD) (Tsai et al., 2016) is the key step of HHT. HHT is employed to assess the time-frequency characteristics in some references (Hopfengärtner et al., 2014; Biju et al., 2017). Hopfengärtner et al. (2014) obtained adaptive energy thresholding in the sub band.

In addition, the applications of Convolutional Neural Network (CNN) toward the detection of epileptic seizures have been implemented. Acharya et al. (2018) found that a 13-layer deep CNN showed an accuracy of 88.67% by using the database of the University of Bonn. The EEG image study based on CNN showed that the true positive rate was 74.0% between seizures and non-seizures EEG activities (Emami et al., 2019). Especially, the research taking time-frequency analysis as the features and combining with CNN is also increasing. The highest classification accuracy of 82.85 and 88.30% was achieved using transfer learning and extract image features approach, respectively, (Raghu et al., 2020). Ansari et al. (2019) achieved the seizure detection rate of 77.0% by using deep CNN with 26 neonates. San-Segundo et al. (2019) used EMD and CNN to classify focal and non-focal signals, which achieved an accuracy of 98.9%.

The above researches are based on the time-frequency domain of multi-channel EEG. Furthermore, multi-channel EEG connectivity in spatial domain is represented by brain networks. With the development of medical imaging technology, more and more evidence shows that some brain diseases, such as epilepsy, Alzheimer’s disease, depression and schizophrenia, have abnormal brain function connections (Bansal et al., 2019). Therefore, researchers’ exploration of the brain has gradually shifted from structural analysis to the functional connections among brain regions. In addition to quantifying and modeling observations in laboratory animals, researchers can perform whole-region simulations of the human brain based on noninvasive imaging data (Lynn and Bassett, 2019). The scalp EEG is more convenient to collect and the cost is lower than other types of data (Lu et al., 2021), so a brain network is built by using scalp EEG in this paper. Transfer entropy (TE) is an information-theoretic measure method originally introduced by Schreiber (2000) to evaluate effective connectivity and it is often used to estimate “information flow” in the brain and analyze EEG signals. The rules defining nodes and edges in association networks are not the same for different medical data. For example, the number of EEG channels, such as 23 channels, 64 channels, 128 channels, etc., determines the number and distribution of network nodes. The calculation methods of the correlation among signals, such as mutual information, TE, phase lock value, Granger causality, Pearson correlation, etc., determine the edge weight of the network. TE is often used to measure the strength of functional connection of neurons (Schreiber, 2000). In this paper, symbolic transfer entropy(STE) based on symbolic dynamics is selected because it is insensitive to signal noise and does not require high parameter coordination (Li et al., 2020).

To research the properties of brain networks, the researchers use the topological properties which include global efficiency, cluster coefficient, average path length, etc. (Wang et al., 2014; Shimono and Beggs, 2015). Besides, the others reveal the dynamic mechanisms of brain network to explain large-scale neural behavior emerging from individual neurons (Lv et al., 2021). Kuramoto model is often used to describe the large-scale neural activity. Majhi et al. (2018) summarized that recent research had shown that the coexistence of coherent and incoherent states, known as chimera states or simply chimeras, is particularly important and characteristic for neuronal systems.

It is clear from the literature that no successful combined studies (in terms of characteristic of epileptic multi-EEG) have been proposed for the multi-channel scalp EEG. Therefore, to explore the characteristics of epileptic EEG signals from the perspective of whole brain were studied by using multi-channel scalp EEG in this paper. Our research work was carried out from the following: firstly, the 1D signals were converted into HS images stack, then, the concatenated images were fed into CNN. Secondly, the 23 channels signals were converted into brain networks by STE among different channels EEG. Thirdly, the networks properties and synchronous behavior by brain network analysis toolbox and Kuramoto model in which the coupling matrix was the above networks were observed. The results show that compared to the previous approach, these methods achieve comparable identification results, besides, our research method can provide effective physical markers for epileptic seizures.

## 2. Methodology

### 2.1. Hilbert-Huang transform

HHT can reflect the energy information of multi-channel EEG in time-frequency domain. HHT is a method composed of EMD and Hilbert Transform (HT). The signal is adaptively decomposed into different IMFs by EMD, and then each IMF is transformed by HT. EMD is a decomposition method to generate IMFs by repeatedly averaging the envelope of maximum and minimum values (Huang et al., 1998). It can be decomposed directly without prior analysis and research for an unknown signal. This method automatically divides the signal according to some fixed modes and levels without manual setting and intervention. The original signal can be obtained by EMD decomposition.

The analytic signal of a single frequency component signal can be obtained through HT, assuming that the analytic signal z(t) is:


(1) $$z\left(t\right)=c\left(t\right)+jy\left(t\right)=a\left(t\right)e^{j\theta \left(t\right)}$$

$a\left(t\right)=\sqrt{c{\left(t\right)}^{2}+y{\left(t\right)}^{2}}$ represents instantaneous amplitude. $\theta \left(t\right)=\arctan\frac{y\left(t\right)}{c\left(t\right)}$ represents instan-taneous phase. $\omega \left(t\right)=\frac{d\theta \left(t\right)}{dt}$ represents instantaneous frequency. The signal can be expressed as:


(2) $$x\left(t\right)=a\left(t\right)e^{j\int \omega \left(t\right)dt}$$

If ${\left|a\left(t\right)\right|}^{2}$ is used as the instantaneous energy, the instantaneous energy distribution of the signal can be drawn on the time-frequency plane, and this distribution spectrum is Hilbert Spectrum (HS) which is marked as H(ω,t) . The 1D original signal is refined into different components and expanded into the 2D image by HHT. The scale of data will be expanded from the dimensions of time, phase and frequency domain. According to its frequency, amplitude and physiological characteristics, the EEG signal with conventional bands includes *α*(8 ~ 13 Hz), *β*(14 ~ 30 Hz), *θ*(4 ~ 7 Hz), *δ*(0.5 ~ 3 Hz).

HS can reflect the energy distribution of different frequency bands. The HS presents the amplitude, instantaneous frequency and time of the original wave simultaneously. In wave dynamics, the squared amplitude is frequently used to represent the energy density of the original wave, hence, the HS represents the Hilbert energy of the original wave. Hilbert Marginal Spectrum(HMS) is the integral of HS in time. From the perspective of integration, all amplitudes in time are added up for any first order frequency to reflect the amplitude accumulation of each frequency in all times and reflect the relationship between the frequency and amplitude of the signal. The HMS offers a measure of total energy contribution from each frequency value and corresponds to energy density at frequency *f*. The HMS represents the cumulated energy of the EEG over the entire data span in a probabilistic sense (Fu et al., 2015).

### 2.2. Symbolic transfer entropy

*TE* is a parameter that measures the degree of correlation between two time sequences. Because *TE* is based on the transition probability and is asymmetric, it mixes directional and dynamic information. *TE* is defined as follows (Staniek and Lehnertz, 2008).


(3) $$TE_{J\to I}=\sum p\left(i_{n+1},i_{n}^{\left(k\right)},j_{n}^{\left(l\right)}\right)\times \log\frac{p\left(i_{n+1}|i_{n}^{\left(k\right)},j_{n}^{\left(l\right)}\right)}{p\left(i_{n+1}|i_{n}^{\left(k\right)}\right)}$$

$i_{n}$ , $j_{n}$ represent the state of sequence *I* and *J* at time *n* respectively, $i_{n}^{\left(k\right)}$ refers to a string of length k , $i_{n-k+1},\cdots ,i_{n}$ ，similarly, $j_{n}^{\left(l\right)}$ refers to a string of length l , $j_{n-l+1},\cdots ,j_{n}$ . The *TE* of *J* to *I* is information flow transferred from *J* to *I*, which can be used as an indicator of causality.

The above-mentioned *TE* is more sensitive to noise, so STE which has the advantage of being insensitive to noise and is more suitable for non-stationary continuous time series is employed (Staniek and Lehnertz, 2009). Providing symbolic sequence of signal $SI{}_{n}$ and $SJ{}_{n}$ , the STE can be calculated as:


(4) $$STE_{J\to I}=\sum p\left(SI{}_{n+1},SI{}_{n},SJ{}_{n}\right)\log\frac{p\left(SI{}_{n+1}SI{}_{n},SJ{}_{n}\right)}{p\left(\frac{SI{}_{n+1}}{SI{}_{n}}\right)}$$

### 2.3. Brain networks and Kuramoto model

Because TE is directional, the brain network constructed is a positive and negative coupling network. Based on these studies, we use STE to build a brain functional network. The network is a weighted directed network with the characteristics of time, structure and direction.

The nodes correspond to different channels and the edge weight is the value of STE. The brain network is treated as a coarse-grained representation of neuron cluster network, which is used as the coupling matrix of the coupled dynamic equation to find out the synchronous behavior of the neuron cluster. To facilitate the simulation of the synchronous behavior of these networks, the Kuramoto model is used as a simplified neural mass model to provide the basis for testing the synchronization of the neural oscillation (Rodrigues et al., 2016; Ma and Tang, 2017). The Kuramoto model is as follows:


(5) $$\frac{d\theta_{i}}{dt}=\omega_{i}+\frac{K}{N}\sum_{j=1}^{N}G_{ij}\sin\left(\theta_{j}-\theta_{i}\right)$$

Where $\theta_{i}$ and $\theta_{j}$ are the phase of the *i-*th and *j-*th oscillator, $\omega_{i}$ is intrinsic frequency, and *K* is the coupling constant, *G_ij_* is coupling matrix which represents an *N × N* matrix with *N* = 23, the reason is that the EEG signals in the dataset in this paper have 23 channels.

### 2.4. Electroencephalograph signals dataset and processing

**Figure 1:**
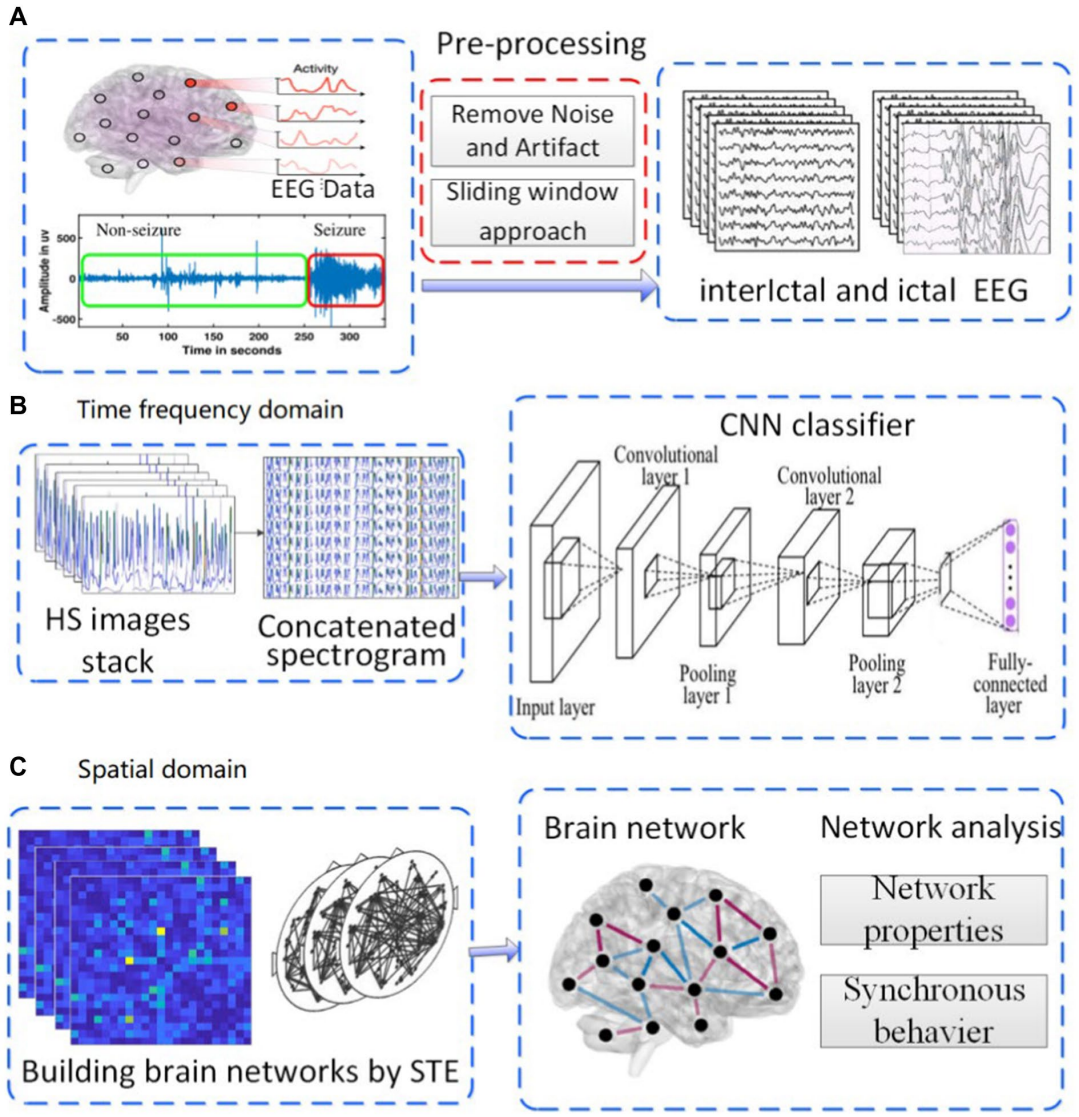
Experimental roadmap. (**A**) pre-processing of EEG. (**B**) analysis in time-frequency domain. (**C**) analysis in spatial domain.

CHB-MIT dataset is the EEG signals from Children’s Hospital of Boston (CHB) included in the Massachusetts Institute of Technology (MIT) EEG database. The EEG data with the sampling frequency of 256 Hz are taken from the open dataset collected by a team of investigators from CHB-MIT^1^ (Shoeb, 2009). This dataset contains scalp EEG records of 22 epileptic patients (5 males, 3 to 22 years old, 17 females, 1.5 to 19 years old). These EEG signals are recorded for 1 h using the international 10–20 EEG electrode position and naming system. Most EEG signals files contain 23 channels in this dataset. In each file containing the data of the seizure that has occurred, the dataset of the beginning and end of 182 seizures are annotated. We divided the one-hour EEG signals into multiple segments of 10 s, and separated the inter stages from the interictal stage state. Figure 1 shows the flow chart of the preprocessing method, which includes EEG signal preprocessing, feature extraction, and classification of interictal and ictal states to detect seizures. The ictal signals contain many types of abnormal waveforms and their amplitude and frequency have changed greatly.

It is well known that the above CHB-MIT dataset is scalp EEG dataset, which contains a lot of noise and artifacts, including blink artifact, eye movement artifact, myoelectricity interference, electrocardio interference, power frequency interference, amplifier saturation, pulse interference, etc. It is necessary to clean up these interference signals before studying the EEG signals. Thus, after comparing and analyzing various EEG processing tools, a new tool based on Python-MNE library, namely MNELAB is selected (Gramfort et al., 2013). The Python-MNE library is one of the python libraries designed to deal with EEG specifically. The preprocessing method in this paper is to use the MNELAB tools for commonly denoising in EEG signals by using frequency limiting and fast Independent Component Analysis (ICA) (Antony et al., 2022).

After preprocessing and screening, and reference to previous literature(Acharya et al., 2018; Emami et al., 2019), we finally extracted 2,500 s interictal EEG and 2,500 s ictal EEG from the dataset. Then, a total of 5,000 s EEG signals with 23 channels were split into segments of 10s each and then converted them into HS images stack. Therefore, 500 EEG segments were generated, which contained 250 interictal and 250 ictal EEG segments.

## 3. Results and discussion

To study the characteristics of epileptic EEG signals from the perspective of the whole brain，this paper proposed an approach of multi-channel characteristics from time-frequency and spatial domains. Thus in this paper, the experimental scheme was carried out in the following three steps shown in Figure 1.

(i) The original EEG signals were preprocessed according to the following steps: Firstly, the background noise and artifact in original signal were removed. Denoised signals were split into segments of 10s each (Figure 1A). Secondly, the processed signals were converted into HS images, then, in a 23 channels EEG signal segment, HS images stack was concatenated into a single spectrogram. Thirdly, the concatenated images were used as the input layer of CNN classifier to identify the ictal EEG (Figure 1B). (ii) The brain function network was constructed by using the processed EEG signals, information transfer among different channels was investigated by using the network, and the network properties were calculated(Figure 1C). (iii) According to the mean field theory, the whole neural networks could be coarse-grained into network of brain regions. The Kuramoto model was used to study the synchronous behavior of these networks.

### 3.1. Seizure identification by HS and CNN

**Figure 2:**
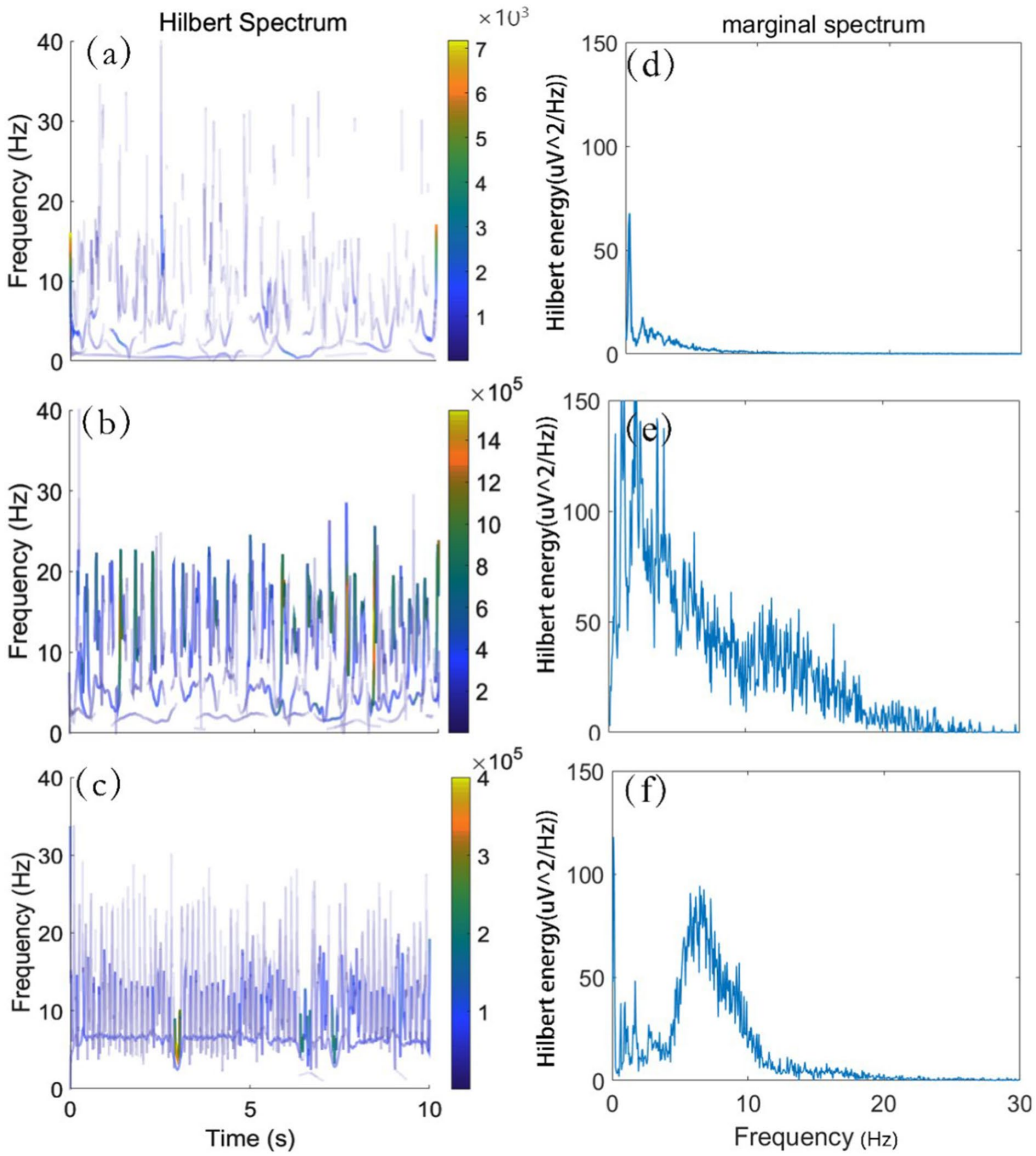
HS and HMS of ictal and interictal EEG. **(A)** HS of interictal EEG. **(B,C)** HS of ictal EEG. **(D)** HMS of interictal EEG. **(E,F)** HMS of ictal EEG.

The HS reflects the instantaneous frequency and amplitude and the energy distribution characteristics of the signals in time-frequency domain, while the HMS represents the energy contribution from each frequency value. Some studies have reported that analyzing EEG signals in the frequency domain could be used effectively for subsequent pattern recognition tasks. Inspired by these results, the recorded EEG time series signal into HS images which reflected the Time-frequency characteristics were transformed in our paper. To observe the EEG time-frequency characteristic, HS and HMS images of three segments of single channel signals were selected and plotted as shown in Figure 2. In addition, we selected signals in other time periods for processing for many times, and similar phenomenon occurred.

One can see from Figures 2A–C are HS images, its abscissa, ordinate represent time, frequency, respectively. To facilitate observation, we intercepted the effective frequency range of 0-40 Hz. By comparing Figures 2A–C, we found that the energy distribution of HS image in interictal phase was more dispersed and smaller energy value than that in ictal phase. At the same time, the HMS images corresponding to the above three signals were depicted in Figures 2D–F, in which the abscissa and the ordinate described the frequency and the energy amplitude, respectively. The HMS offers a measure of total HE contribution from each frequency value. The area below the HMS curves in Figures 2D–F represents the total HE over the entire frequency span. It can be observed from HMS that the energy of interictal EEG is contained mostly in δ band, while the δ band in the ictal EEG accounts for a small proportion of total energy.

To further study the feasibility of HS in automatic seizure identification, we converted the EEG signal segments into HS images and concatenated the images into an image stack (Figure 1B). The CNN classifier was used for automatic identification of the above concatenated images.

1. To obtain more information of the same time segment of EEG, 1D signals were converted into 2D concatenated images.
2. To overcome the imbalance issue of CNN, the same duration of interictal and ictal EEG signals were extracted, and interictal EEG signals from the large number of interictal phases were extracted randomly.
3. To identify epileptic EEG signals accurately, two consecutive sets of convolutional/pooling layers were used. Convolutional layer could extract edges, shapes and textures of a spectrogram. The activation function of convolutional layers was ReLU. The convolution filter size was 3 × 3 and the number of convolutional units was 32 and 64, respectively. We adopted the maxpooling layer, set the pooling size to 2 × 2, and used Adam as the optimizer which solved the problem of large swing range in optimization and can speed up the convergence of function. Two layers of pooling layer and one layer of dropout layer were designed, which was to reduce the model complexity while retaining key information, prevented overfitting of the model and improved the generalization ability of the model. The cross-validation was leave-one-out cross validation(LOOCV). 80% data were selected for the training set, and the rest data were used for the test one.
4. To verify generalizability of the model, external EEG signals of other subjects (except the 10 subjects) in CHB-MIT dataset were used to test.

Sensitivity, accuracy, AUC (area value under ROC curve), F1_score and specificity were calculated to evaluate the performance of the classifier, as shown in Table 1. The identification effect of 96.5% was also achieved for the external images. We summarized the references of EEG detection and classification of epilepsy using time-frequency spectral analysis or machine learning. Our results and the comparative classification result are listed in Table 1.

**Table 1:** Seizure identification results and comparison table of classification results (Sen, Sensitivity; Acc, Accuracy; Spe, Specificity).

Authors	Methods	EEG data source	Performance (%)
Acharya et al. (2018)	13-layer deep CNN structure	Bonn	Acc: 88.4
Mandhouj et al. (2021)	Using STFT Spectrogram with deep CNN	Bonn	Acc: 98.22
Yuan et al. (2019)	Spectrogram with STFT using multi-view deep learning framework	CHB-MIT	Acc: 94.3
			AUC: 95.7
Tsiouris et al. (2018a)	Discrete wavelet transform (DWT) + LSTM	CHB-MIT	Sen: 99.84
			Spe: 99.86
Fergus et al. (2015)	Power spectral density (PSD) + KNN	CHB-MIT	Sen: 95.1
Tsiouris et al. (2018b)	Spectral analysis, STFT+SSM	CHB-MIT	Sen: 88
Rashed-Al-Mahfuz et al. (2021)	VGG16+ frequency components	CHB-MIT	Acc: 99.2
Our paper	Spectrogram with HHT of multi-channel EEG using CNN	CHB-MIT	Sen: 99.8
			Spe: 97.4
			Acc:98.7
			AUC:99.9
			F1_score:98.7

From Table 1 one notice that some recent research methods could achieve a certain degree of classification effect. However, our proposed scheme using multi-channel scalp EEG automatic recognition could obtain better classification effect. Compared with the exiting studies, it was found that our model achieves comparable identification effect. It showed that it was feasible to identify seizures from the perspective of whole brain.

### 3.2. Building brain networks

The above method can expand the energy distribution of epileptic EEG signals and identify epileptic signals more accurately in time-frequency domain. However, in spatial domain, because the brain network constructed by 23 channels EEG can more truly reflect the information transmission and functional activities among the brain regions, it is also very important to further analyze the causality and connectivity among different parts of the brain. Next, EEG signals are used for further research the synchronous behavior of the brain networks.

**Figure 3:**
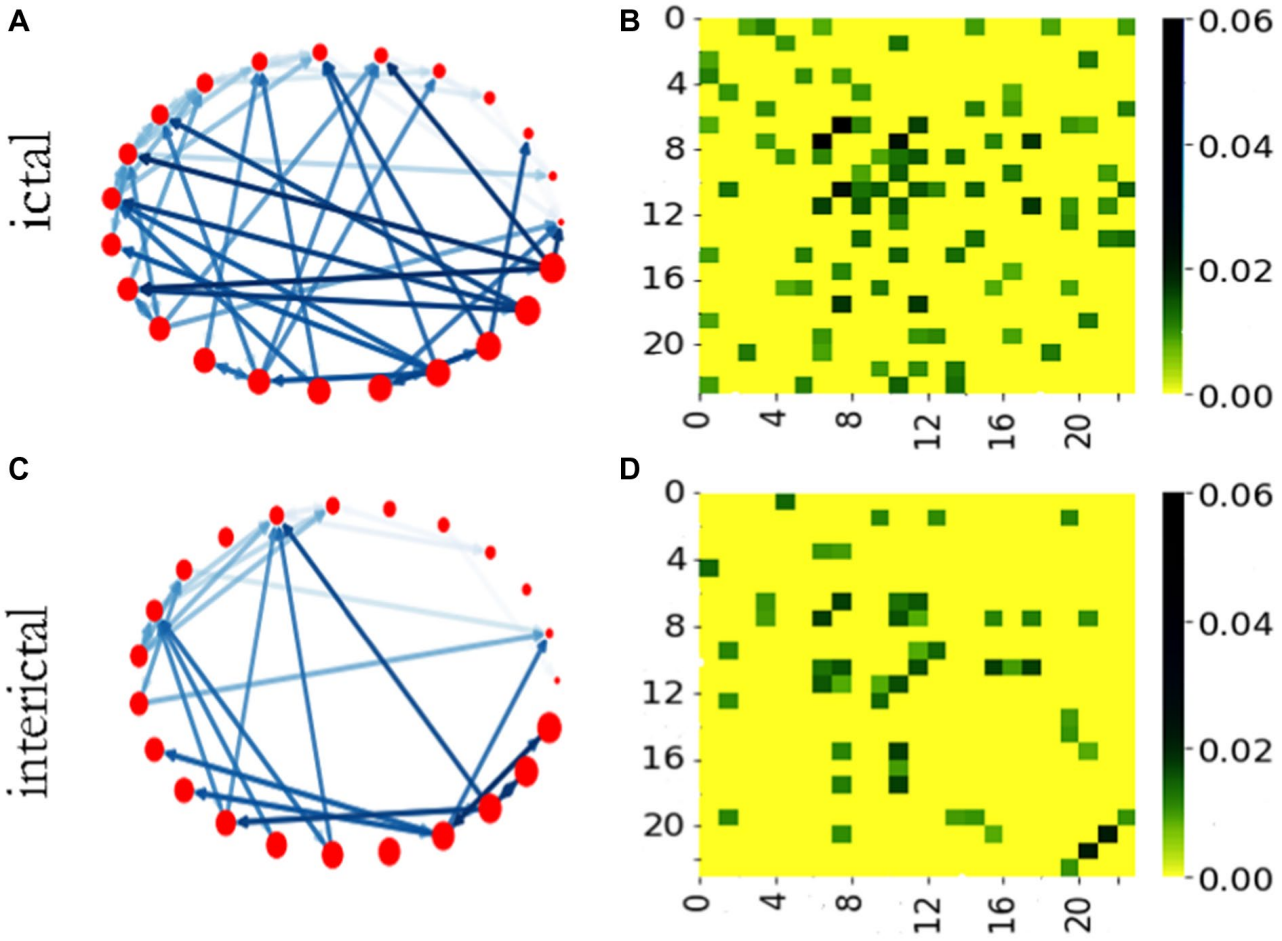
Brain networks and adjacency matrices of ictal and interictal phases. **(A)** The network in ictal stage. **(B)** Adjacency matrix of ictal network. **(C)** The network in interictal stage. **(D)** Adjacency matrix of interictal network.

We calculated the STE values among each signal segment, and built a brain function network of 23 nodes with STE as the edge weight. Then, using this method, a weighted adjacent network with 500 multi-channel signal segments in batch was built. The representative diagrams of network and adjacency matrix are shown in Figure 3. We set the threshold by traversal and found the appropriate threshold, and set the threshold of STE to 0.02 after verification in the subsequent experiment. For clearer image display, we set the threshold of STE to 0.08. Only edges larger than the threshold value could be drawn.

Figures 3A,C show brain functional networks of ictal and interictal phases. The different colors of the network connection edges represent the relative intensity of the STE, the network nodes correspond to the channels, and the arrows indicate the directions of “information flow.” Therefore, this brain network is a weighted network with direction, which may provide some useful clues for the localization of epileptic focus. Figures 3B,D show adjacency matrices of ictal and interictal phases. The vertical and horizontal coordinates of the adjacency matrix heat map represent the number of the network node, and the color bar describes the value of the STE. We can see from this figure, under the same threshold conditions, the network has more connectors during ictal phase than during interictal phase in most cases.

### 3.3. Network analysis

**Figure 4:**
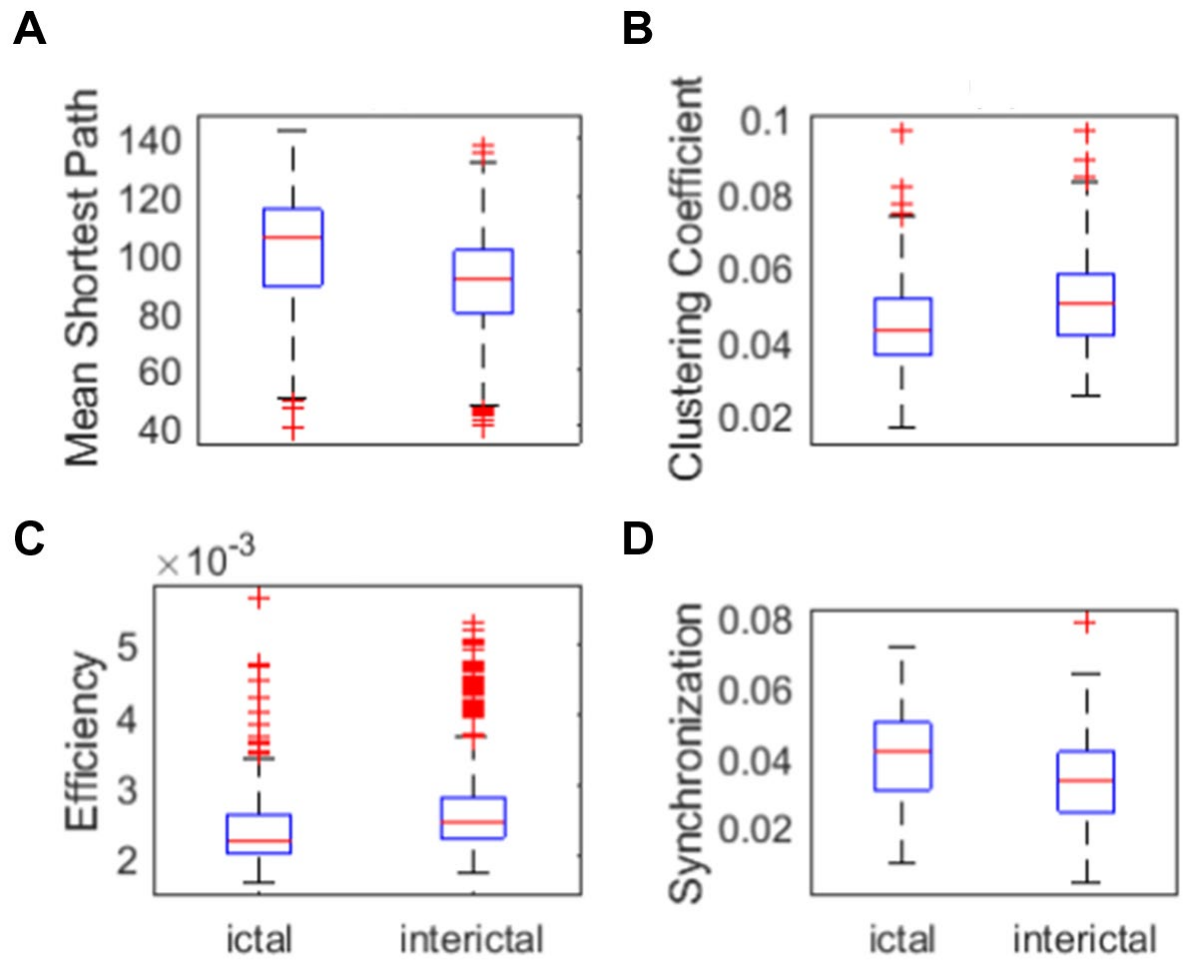
Network properties. **(A)** Mean shortest path of ictal and interictal networks. **(B)** Clustering coefficient of ictal and interictal networks. **(C)** Efficiency of ictal and interictal networks. **(D)** Synchronization of ictal and interictal networks.

To verify the feasibility and effectiveness of our methods, the following two schemes were adopted: one was to use network analysis toolbox, the other one was Kuramoto phase oscillator model. As the first test method, the analysis toolbox called GRETNA^2^ (Wang et al., 2015) which was a graph theoretical network analysis toolbox for imaging connectomics was adopted. Network properties of the 500 networks constructed above was calculated. Some network properties parameters, such as mean shortest path, clustering coefficient, efficiency and synchronization were used to distinguish the ictal and the interictal phases. The results are shown in Figure 4.

One can see from Figure 4A that, the mean shortest path in ictal phase is greater than that in interictal phase. Clustering coefficient in the ictal phase is lower than that in the interictal phase(Figure 4B). Efficiency of ictal networks is lower than efficiency of interictal networks (Figure 4C), which represents the work efficiency of the brain decline during the seizure. Clinical studies show that patients with intractable epilepsy often have cognitive impairment, including memory loss, language, expression problems, and intellectual decline. According to the graph theory, the brain efficiency of patients with refractory epilepsy is lower, suggesting that the efficiency of long-distance information transmission and the ability to integrate information of patients with refractory epilepsy are reduced within a certain range. Therefore, mean shortest path in ictal stage is higher. Clustering coefficient is lower in ictal stage due to shorter path length can promote clustering of network node. The efficiency in ictal stages is lower due to the impact on brain function during the seizures. These clinical conclusions are consistent with the experimental results.

As can be seen in Figure 4D, synchronization in ictal phase is greater than that in interictal phase, which represent the cerebral cortex during ictal phase is more susceptible to abnormal synchronous discharges. Next, the synchronization of the brain network is further verified. We performed some statistical analysis to determine the difference of parameters of network properties between ictal signal and interictal signal. This was confirmed by the lack of concordance between statistical analysis and main part of the paper. Therefore, we proposed not mentioning these analyzes.

The second verification method is to use Kuramoto phase oscillator model. As a simplified neural quality model, this model can be used to describe the average field of large-scale neural activities, so as to further verify the synchronization of multi-channel EEG brain network and explain the large-scale neuroelectrical behavior of a single neuron. In the network, STE was used as the weight of edges, the greater the STE, the stronger the information transfer intensity between the two channels and the closer the connection between the two nodes.

**Figure 5:**
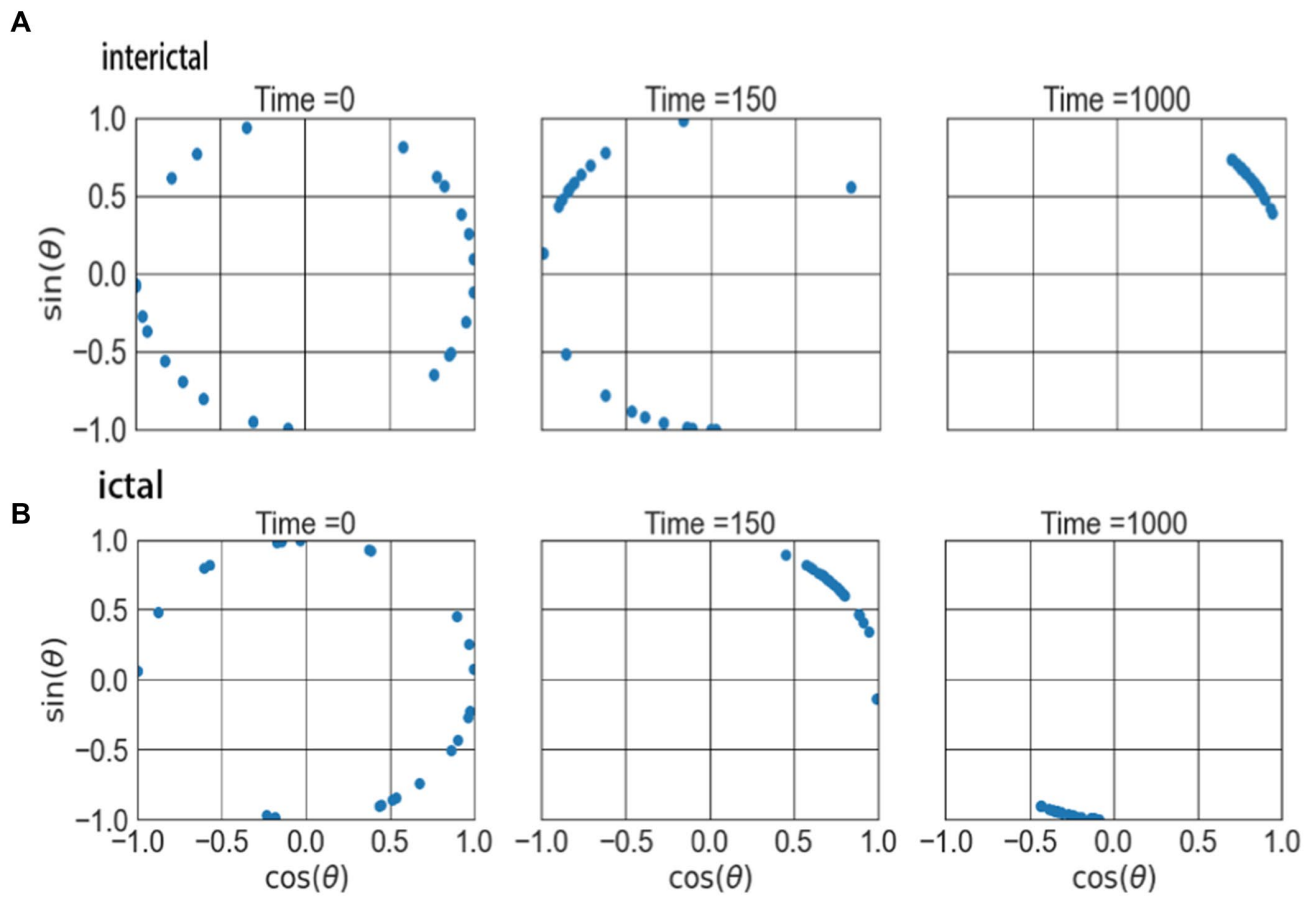
The attractors of two type of networks on the complex plane. **(A)** The attractors of interictal network. **(B)** The atttactors of ictal network.

In order to observe the attractor synchronous behavior in the process of time evolution better, networks of ictal and interictal phases with similar coordination coefficients are selected. In the model, the internal frequencies of 23 coupling oscillators are evenly distributed in the interval [0,1], the coupling strength *K* = 3.5. The results are shown in Figure 5.

Figure 5 shows the evolution result of the 23 oscillators over time. At the beginning, the phases of these oscillators are different and randomly distributed in different positions on a ring of the network, as shown in the first column of the figure. As time goes on, the oscillators begin to gather in one direction, and when *t* = 150, the oscillators have contracted and converged to a certain extent (as shown in the middle column). When the time evolution reaches *t* = 1,000, the oscillators further shrink and converge to form an attractor structure, as shown in the right column in the figure. This indicates that the signals during the ictal enter the synchronization state more quickly. These network properties can provide reference for exploring the non-invasive identification marks and dynamic mechanisms of epilepsy.

## 4. Conclusion

In the paper, we explored the characteristics of EEG signals in ictal and interictal phases in time-frequency and spatial domain. HS reflect time-frequency characteristics of multi-EEG in time frequency domain, and achieve good identification results. The sensitivity is 99.8%, accuracy is 98.7%, specificity is 97.4%, F1-score is 98.7%, and AUC-ROC is 99.9%. Brain function networks which reflect spatial characteristics of multi-EEG present different characteristics between ictal and interictal phase, which is verified by network properties and Kuramoto model. Experiments indicates that the network properties are different between interical stages and ictal stages, and the signals during the ictal enter the synchronization state more quickly. This part of work can also be improved from the following two aspects: (1) Due to the less number of scalp EEG channels in this data set, the function network could not describe the details of EEG signals. (2) Some networks did not support the experimental results with Kuramoto model. The next step is to further research the relationship between network hierarchy and remote synchronization or relay synchronization, which requires the construction of more complex brain networks, such as EEG with more channels, MEG, and fMRI networks as well.

## Data availability statement

The original contributions presented in the study are included in the article/supplementary material, further inquiries can be directed to the corresponding author.

## Author contributions

XL: investigation, methodology, methodology development or design of methodology, visualization writing–original draft, writing–review and editing, and data curation. JZ: conceptualization, methodology, funding acquisition, project administration, supervision, and writing-review and editing. SH: formal analysis, validation, and software. MY: data collection and validation. MW and TW: data analysis and processing. All authors contributed to the article and approved the submitted version.

## Funding

This study was funded by the project of University Natural Science Research Project of Anhui Province (No. KJ2020A0618), the project of Academic and technical leaders candidate of Anhui Province (No. 2022H286), the Natural Science Foundation of Anhui Province, China (No. 1908085MA25), the Key Research and Development Plan of Anhui Province, China (No. 2022a05020011), the University Synergy Innovation Program of Anhui Province, China (No. GXXT-2022-044), the Excellent Scientific Research Innovation Team Project of Universities in Anhui Province, China (No. 2022AH010075), and the Academic Support Project for Top-notch Talents in Disciplines (Majors) of Universities in Anhui Province, China (No. gxbjZD2022042).

## Conflict of interest

The authors declare that the research was conducted in the absence of any commercial or financial relationships that could be construed as a potential conflict of interest.

## Publisher’s note

All claims expressed in this article are solely those of the authors and do not necessarily represent those of their affiliated organizations, or those of the publisher, the editors and the reviewers. Any product that may be evaluated in this article, or claim that may be made by its manufacturer, is not guaranteed or endorsed by the publisher.

## Footnotes

^1^ https://www.physionet.org/content/chbmit/1.0.0/

^2^ http://www.itrc.org/projects/gretna/
