# Corrigendum: Study on characteristic of epileptic multi-electroencephalograph base on Hilbert-Huang transform and brain network dynamics

**DOI:** 10.3389/fnins.2023.1221328

**Published:** 2023-05-25

**Authors:** Xiaojie Lu, Tingting Wang, Mingquan Ye, Shoufang Huang, Maosheng Wang, Jiqian Zhang

**Affiliations:** ^1^School of Physics and Electronic Information, Anhui Normal University, Wuhu, China; ^2^Research Center of Health Big Data Mining and Applications, School of Medicine Information, Wan Nan Medical College, Wuhu, China

**Keywords:** Hilbert-Huang transform, CNN, symbolic transfer entropy, brain network, Kuramoto model

**Incorrect Funding**

In the published article, there was an error in the Funding statement. The Key Research and Development Plan of Anhui Province, China (NO. 2022a05020011), the University Synergy Innovation Program of Anhui Province, China (NO. GXXT-2022-044), the Excellent Scientific Research Innovation Team Project of Universities in Anhui Province, China (NO. 2022AH010075), and the Academic Support Project for Top-notch Talents in Disciplines (Majors) of Universities in Anhui Province, China (NO. gxbjZD2022042). The correct Funding statement appears below.

